# L-citrulline production by metabolically engineered *Corynebacterium glutamicum* from glucose and alternative carbon sources

**DOI:** 10.1186/s13568-014-0085-0

**Published:** 2014-12-10

**Authors:** Dorit Eberhardt, Jaide V K Jensen, Volker F Wendisch

**Affiliations:** Genetics of Prokaryotes, Faculty of Biology & Center for Biotechnology, Bielefeld University, Universitätsstraße 25, Bielefeld, 33615 Germany

**Keywords:** Corynebacterium glutamicum, L-citrulline, L-arginine, Alternative carbon sources, Starch, Xylose, Glucosamine, Metabolic engineering

## Abstract

L-citrulline plays an important role in human health and nutrition and is an intermediate of the L-arginine biosynthetic pathway. L-citrulline is a by-product of L-arginine production by *Corynebacterium glutamicum*. In this study, *C. glutamicum* was engineered for overproduction of L-citrulline as major product without L-arginine being produced as by-product. To this end, L-arginine biosynthesis was derepressed by deletion of the arginine repressor gene *argR* and conversion of L-citrulline towards L-arginine was avoided by deletion of the argininosuccinate synthetase gene *argG*. Moreover, to facilitate L-citrulline production the gene encoding a feedback resistant N-acetyl L-glutamate kinase *argB*^fbr^ as well as the gene encoding L-ornithine carbamoylphosphate transferase *argF* were overexpressed. The resulting strain accumulated 44.1 ± 0.5 mM L-citrulline from glucose minimal medium with a yield of 0.38 ± 0.01 g⋅g^−1^ and a volumetric productivity of 0.32 ± 0.01 g⋅l^−1^⋅h^−1^. In addition, production of L-citrulline from the alternative carbon sources starch, xylose, and glucosamine could be demonstrated.

**Electronic supplementary material:**

The online version of this article (doi:10.1186/s13568-014-0085-0) contains supplementary material, which is available to authorized users.

## Introduction

L-citrulline is a natural non-proteinogenic amino acid whose name is derived from watermelon *Citrullus lanatus* (Wada [1930]). In mammalians it serves as a precursor for L-arginine. In contrast to the proteinogenic L-arginine, which is not transferred to the blood stream, when ingested, L-citrulline can be converted to L-arginine, which is then released by the kidney into the blood stream. It is applied in several medical approaches e.g. as a pharmaconutrient (Rimando and Perkins-Veazie [2005]; Curis et al. [2005]).

Currently, biocatalytic and fermentative methods to produce L-citrulline using *Pseudomonas putida* (Kakimoto et al. [1971]; Yamamoto et al. [1974]) or *Bacillus subtilis* strains exist (Okumura et al. [1966]). Additionally, extraction processes from watermelon have been established (Fish [2012]). L-citrulline is an intermediate of L-arginine biosynthesis and accumulates as a by-product of engineered L-arginine producing *Corynebacterium glutamicum* strains (Ikeda et al. [2009]; Schneider et al. [2011]).

*C. glutamicum* is a workhorse for amino acid production and is employed for the annual production of several million tons of L-glutamate and L-lysine (Wendisch [2014]). *C. glutamicum* has been engineered to produce a wide range of bioproducts, such as diamines, carotenoids, terpenes, proteins (Schneider and Wendisch [2010]; Schneider et al. [2012]; Heider et al. [2014a], [b]; Frohwitter et al. [2014]; Kikuchi et al. [2009]; Teramoto et al. [2011]; An et al. [2013]) and the L-glutamate family amino acids L-arginine, L-ornithine, and L-proline (Schneider et al. [2011]; Ikeda et al. [2009]; Georgi et al. [2005]; Blombach et al. [2009]; Jensen and Wendisch [2013]). However, the production of L-citrulline as the only or major product has not been published yet.

**Figure 1:**
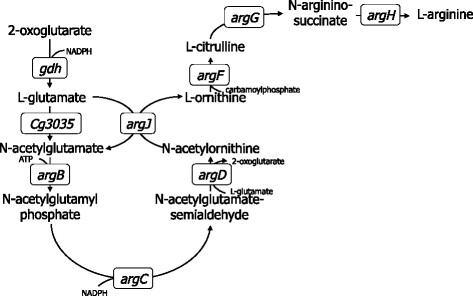
**L-arginine pathway in** ***C. glutamicum*** **(modified from (Wendisch et al.**[2014]**)).** *gdh*: L-glutamate dehydrogenase, *cg3035*: anaplerotic N-acetylL-glutamate synthase, *argJ*: L-ornithine N-acetyltransferase, *argB*: N-acetylL-glutamate kinase; *argC*: N-acetyl-gamma-glutamyl-phosphate reductase; *argD*: acetylL-ornithine aminotransferase; *argE*: acetylL-ornithine deacetylase; *argF*: L-ornithine carbamoyltransferase; *argG*: argininosuccinate synthetase; *argH*: argininosuccinate lyase. Oxoglutarate is an intermediate of the central carbon metabolism.

Due to its natural ability to produce L-glutamate under several eliciting conditions, *C. glutamicum* is a suitable producer of L-glutamate-derived products (Sato et al. [2008]; Radmacher et al. [2005]; Kim et al. [2009], [2010]; Delaunay et al. [1999]; Wendisch et al. [2014]). L-ornithine is a non-proteinogenic glutamate-family amino acid and an intermediate of L-arginine biosynthesis (Figure 1). An ornithine producer was obtained by deletion of *argR*, the gene encoding the genetic repressor of the arginine biosynthesis operon, and *argF* to prevent further processing of ornithine (Schneider et al. [2011]). The production of L-proline from L-ornithine is possible by the heterologous overexpression of *ocd* from *Pseudomonas putida*, encoding ornithine cyclodeaminase (Jensen and Wendisch [2013]). The diamine putrescine can be produced by overexpression of the *Escherichia coli* gene *speC*, which encodes ornithine decarboxylase (Schneider et al. [2012]; Schneider and Wendisch [2010]). As the arginine biosynthetic pathway is naturally regulated by feedback inhibition of N-acetylglutamate kinase (encoded by *argB*) by arginine, the use of feedback resistant enzyme variants in combination with deletion of *argR* has been described to overproduce L-arginine (Sakanyan et al. [1996]; Ikeda et al. [2009]; Schneider et al. [2011]).

**Figure 2:**
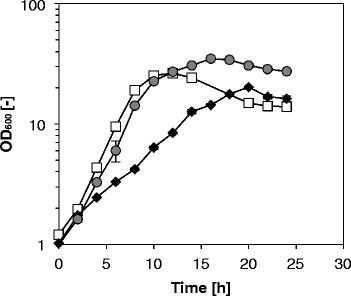
**Biomass formation by various** ***C. glutamicum*** **strains.** The cultivation was performed in CGXII minimal medium containing 20 g L-1 glucose, 1 mM IPTG, 750 μM L-arginine and 25 μg L-1 kanamycin. OD_600_ was determined of CIT0(pVWEx1) (open squares), CIT0(pVWEx1-*argF*) (gray circles) and CIT0(pVWEx1-*argFB*^fbr^) (black diamonds). Values and error bars represent the mean and the standard error of triplicates.

*C. glutamicum* can utilize a variety of carbon sources. In contrast to many other microorganisms used in biotechnology, simultaneous utilization of carbon sources e.g. present in mixtures such as lignocellulosic hydrolysates is a hall mark of *C. glutamicum* (Blombach and Seibold [2010]; Meiswinkel et al. [2013a], [b]). The natural substrate spectrum of *C. glutamicum* includes monosaccharides, disaccharides, and organic acids as well as alcohols (Blombach and Seibold [2010]; Arndt and Eikmanns [2008]; Peters-Wendisch et al. [1998]; Jolkver et al. [2009]; Sasaki et al. [2011]). To allow access to alternative carbon sources, *C. glutamicum* has also been engineered for utilization of glycerol, pentoses, and amino sugars as well as polysaccharides (Schneider et al. [2011]; Rittmann et al. [2008]; Seibold et al. [2006]; Uhde et al. [2013]; Gopinath et al. [2011]; Matano et al. [2014]).

One aim to reduce production cost is the use of complex sugar substrates for the production of biotechnological products. As an example of using a polymeric raw material without decomposition to its monomeric compounds e.g. by enzyme treatment, soluble starch could be used as a carbon source for the production of L-lysine and organic acids by engineered *C. glutamicum* (Seibold et al. [2006]; Tateno et al. [2007]; Tsuge et al. [2013]). However, due to the growing world population and a correlating higher demand for food, biotechnological processes based on non-food derived carbon sources are sought. Xylose is a pentose sugar compound present in the hemicellulosic fraction of agricultural wastes as for example rice straw. Glucosamine, on the other hand, is a constituent of chitin, the second most abundant biopolymer in Nature, which is accessible e.g. from shrimp shell waste accumulating in the food industry. *C. glutamicum* has been engineered to efficiently utilize both xylose and glucosamine as alternative carbon sources for growth and amino acid production (Gopinath et al. [2011]; Meiswinkel et al. [2013a]; Uhde et al. [2013]; Matano et al. [2014]).

In this study, the rational engineering of L-citrulline production by *C. glutamicum* is reported and the concept was extended to production of L-citrulline from the alternative carbon sources glucosamine, xylose, and starch.

## Materials and methods

### Microorganisms and growth conditions

Microorganisms and plasmids used in this study are listed in Table 1. *E. coli* DH5α was used for gene cloning. *C. glutamicum* and *E. coli* strains were routinely grown in lysogeny broth (LB) (10 g L^−1^ tryptone, 5 g L^−1^ yeast extract, 10 g L^−1^ sodium chloride) in 500-mL baffled flasks on a rotary shaker (120 rpm) at 30°C or 37°C. For growth experiments, CGXII minimal medium (Eggeling and Reyes [2005]) was used for *C. glutamicum*. Growth was followed by measuring the optical density at 600 nm using a V-1200 Spectrophotometer (VWR, Radnor, PA, USA). An OD_600_ of 1 corresponds approximately to an estimated cell dry weight of 0.25 g/L.

**Table 1:** **Strains and plasmids used in this study**

***E. coli***		
DH5α	F^−^*thi*-1 *endA* 1 *hsdr* 17(r^−^, m^−^) *supE* 44 ∆*lacU* 169 (Φ80*lacZ*∆M15) *recA* 1 *gyrA* 96 *relA* 1	(Hanahan [1983])
***C. glutamicum***		
MB001	ATCC 13032 with in-frame deletion of prophages CGP1 (cg1507-cg1524), CGP2 (cg1746-cg1752), and CGP3 (cg1890-cg2071)	(Baumgart et al. [2013])
CIT0	MB001 with ∆*argF*, ∆*argG*, ∆*argR*	This study
CIT1	CIT0 carrying the pVWEx-*argFB*^fbr^ vector	This study
**Plasmids**		
pEKEx3	Spec^R^, P_tac_, lacI^q^	(Stansen et al. [2005])
pVWEx1	Kan^R^, P_tac_, lacI^q^	(Peters-Wendisch et al. [2001])
pEC-XT99A	Tet^R^, P_trc_, lacI^q^	(Kirchner and Tauch [2003])
pK19∆*argFR*	Kan^R^, pk19mobsacB with the deletion construct of genes *argFR*	(Schneider et al. [2011])
pK19∆*argG*	Kan^R^, pk19mobsacB with the deletion construct of genes *argG*	This study
pEKEx3- *argB*^fbr^	Spec^R^, pEKEx3 carrying *argB* from *C. glutamicum* ATCC 13032 with amino acid exchanges A49VM54V	(Schneider et al. [2011])
pVWEx1-*argF*	Kan^R^, pVWEx1 carrying *argF* from *C. glutamicum* ATCC 13032	This study
pVWEx1-*argFG*	Kan^R^, pVWEx1 carrying *argF* and *argG* from *C. glutamicum* ATCC 13032	This study
pVWEx1-*argFB*^fbr^	Kan^R^, pVWEx1 carrying *argF* from *C. glutamicum* ATCC 13032 and *argB*^fbr^ from pEKEx3- *argB*^fbr^	This study
pEKEx3-*nagB*	Spec^R^, pEKEx3 carrying *nagB* from *C. glutamicum* ATCC 13032	(Uhde et al. [2013])
pEKEx3-*xylAB*	Spec^R^, pEKEx3 carrying *xylA* from *Xanthomonas campestris* XCC1758 and *xylB* from *C. glutamicum* ATCC 13032	(Meiswinkel et al. [2013a])
pAMY	Tet^R^, pEC-XT99A carrying *amy* from *Streptomyces griseus* IMRU 3570	(Seibold et al. [2006])

When necessary, the growth medium was supplemented kanamycin (25 μg mL^−1^), spectinomycin (100 μg mL^−1^), tetracycline (10 μg mL^−1^), isopropyl β-D-1-thiogalactopyranoside (IPTG) (1 mM) and L-arginine (750 μM). The growth behavior and L-citrulline production of recombinant *C. glutamicum* strains were analyzed in 500 ml baffled flasks. Briefly, a 50 mL BHI (37 g L^−1^) seed culture was inoculated from an agar plate and grown overnight. The cells were harvested by centrifugation (4,000 × g, 10 min) and washed twice with CGXII minimal medium lacking the carbon source. Subsequently, 50 mL CGXII medium, containing a given concentration of carbon source and necessary supplements, was inoculated to an optical density of 1.0. Detailed information on the carbon source concentrations employed are given in the Results chapter.

### Molecular genetic techniques

Standard methods such as restriction digestions, and ligation were carried out as described elsewhere (Sambrook and Russell [2012]). Digested DNA was purified by using the QIAquick Gel Extraction Kit (Qiagen, Hilden, Germany). *E. coli* cells were transformed by heat shock (Sambrook and Russell [2012]) and *C. glutamicum* cells were transformed by electroporation (Eggeling and Reyes [2005]). Isolation of genomic DNA was performed as previously described (Jensen and Wendisch [2013]). Chromosomal changes in *C. glutamicum* were performed as described elsewhere (Eggeling and Reyes [2005]).

### Construction of strains and plasmids

The deletion of ∆*argFR* in MB001 was performed by using pK19mob*sacB*∆*argFR*. Afterwards *argG* was deleted by using pK19mob*sacB*∆*argG* to obtain CIT0. pK19mob*sacB*∆*argG* contains the up- and downstream regions of *argG* in the ∆*argFR* strain. The plasmid was constructed by amplifying the upstream region with *argG* _up_f (CTT*gaattc* AGAAGCTGCGCCGCATG) and *argG* _up_r (agagacgacctaagccagtctAACGATGCGGTTAGTCATGAGG) and the downstream region with *argG* _down_f (agactggcttaggtcgtctctGCTAACAAGCGCGATCGC) and *argG* _down_r (CCT*ctgcag* AACGACCAGCGCGCAGA). The two fragments were combined by crossover PCR using *argG* _up_f and *argG* _down_r and finally cloned into pK19mob*sacB* with *Pst* I and *Eco* RI.

pVWEx1-*argF* was constructed by amplifying *argF* with primers argF_f (CTT*gtcgac* AAGGAGATATAGATATGACTTCACAACCACAGGTTCG) and argF_r (CCT*ggatcc* TTACCTCGGCTGGTTGGC). The PCR product was treated with *Sal* I and *Bam* HI and ligated with similarly treated pVWEx1. pVWEx1-*argFG* was constructed by amplifying *argG* with primers argG_f (GGG*gtcgac* GAAAGGAGGCCCTTCAGATGACTAACCGCATCGTTCTTG) and argG_r (GGG*gtcgac* TTAGTTGTTGCCAGCTTCGCGA). The PCR product was treated with *Sal* I and ligated with similarly treated pVWEx1-*argF*.

The plasmid vector pEKEx-*argB*^fbr^ (*argB*_A49VM54V_ (Schneider et al. [2011])) was digested with *Bam* HI and *Kpn* I and the DNA fragment with a size of 0.9 kb harboring the *argB*^fbr^ gene was cloned into the *Bam* HI/*Kpn* I digested vector pVWEx1-*argF*.

### Determination of amino acid and carbohydrate concentrations

For the quantification of extracellular amino acids and carbohydrates, a high-performance liquid chromatography system was used (1200 series, Agilent Technologies Deutschland GmbH, Böblingen, Germany). Samples were withdrawn from the cultures, centrifuged (13,000 × g, 10 min), and the supernatant used for analysis.

Glucose and xylose were analyzed on a normal phase column (organic acid resin 300 × 8 mm, 10 μm particle size, 25 Å pore diameter; Chromatographie Service GmbH, Langerwehe, Germany) using 5 mM sulfuric acid as the mobile phase at a flow rate of 1 mL min^−1^ and were detected with a refractive index detector (RID G1362A, 1200 series, Agilent Technologies). Amino acids were automatically modified by precolumn derivatisation with ortho-phthalaldehyde and separated as described previously (Georgi et al. [2005]). L-ornithine was quantified using a pre-column (LiChrospher 100 RP18 EC-5 μ (40 × 4 mm), CS-Chromatographie Service GmbH, Langerwehe, Germany) and a reversed phase column (LiChrospher 100 RP18 EC-5 μ (125 × 4 mm), CS Chromatographie) as a main coulumn and detected with a fluorescence detector at excitation at 230 nm and 450 nm emission (FLD G1321A, 1200 series, Agilent Technologies). For the determination of L-citrulline, a reverse-phase (RP) LiChrospher 100 RP8 EC-5 μ precolumn (40 × 4.6 mm) and a RP8 EC-5 μ (125 × 4.6 mm) main column (CS Chromatographie, Langerwehe, Germany) were used. 100 μM L-Asparagine was used as an internal standard. The mobile phases used were in case of RP8 A: 0.25% Na-acetate pH 6, B: methanol. The gradient used was: 0 min 30% B, 1 min 30% B, 6 min, 70% B, 11 min 90% B, 14 min 70% B, 16 min 30% B. In case of RP18, the mobile phases used were A:0.1 M Na-acetate pH 7.2, B: methanol. The gradient used was: 0 min 20% B, 0.5 min 38% B, 2.5 min 46% B, 3.7 min 65% B, 5.5 min 70% B, 6 min 75% B, 6.2 min 85% B, 6.7 min 20% B.

## Results

### Engineering a prophage-free *C. glutamicum* strain for L-citrulline production

*C. glutamicum* has recently been cured of prophage sequences to yield MB001 (Baumgart et al. [2013]). This strain was used as the parental strain because it can be transformed easily and plasmid-based gene overexpression is more efficient (Baumgart et al. [2013]). As *C. glutamicum* ATCC 13032, this strain does not accumulate L-citrulline, an intermediate of L-arginine biosynthesis (Figure 1). The deletion of three genes of the L-arginine operon (L-ornithine carbamoyltransferase (EC 2.1.3.3) *argF*, argininosuccinate synthetase (EC 6.3.4.5) *argG*, and L-arginine biosynthesis operon repressor gene *argR*) in *C. glutamicum* MB001 yielded the L-arginine auxotrophic strain CIT0 (Table 1). When supplemented with 0.75 mM L-arginine, *C. glutamicum* CIT0 accumulated 25.2 ± 2.6 mM L-ornithine from 2% glucose (Table 2). The deletion of *argF* and *argG* could be complemented by plasmid-borne expression of these genes since the complemented strain CIT0(pVWEx1-*argFG*) grew without L-arginine supplement while the empty vector carrying control CIT0(pVWEx1) did not (data not shown). Comparable growth rates and biomass concentrations were observed.

**Table 2:** **Growth on different carbon sources**

***C. glutamicum***strain	Carbon source concentration	Maximum OD_600_	Growth rate (h^−1^)
CIT1(pEKEx3-*xylAB*)	Xylose: 15 g/L	6 ± 1	0.03 ± 0.01
CIT1(pEKEx3-*nagB*)	Glucosamine: 10 g/L	3 ± 1	0.02 ± 0.01
CIT1(pAMY)	Soluble starch: 10 g/L Glucose: 2.5 g/L	9 ± 1	0.21 ± 0.01
CIT1(pEC-XT99A)	Soluble starch: 10 g/L Glucose: 2.5 g/L	3 ± 1	0.10 ± 0.01

Fermentations were performed in CGXII minimal medium containing the respective carbon source and were supplemented by 750 μM L-arginine. 1 mM IPTG and 25 μg/ml kanamycin and spectinomycin were added. Values and error bars represent the mean and the standard error of triplicates.

**Figure 3:**
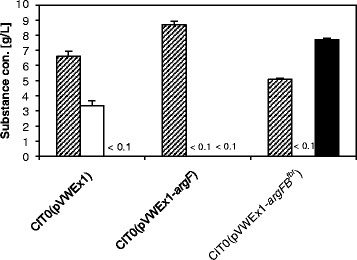
**Biomass formation and production of ornithine and citrulline on glucose by various** ***C. glutamicum*** **strains: cell dry weight (hatched bars), L-ornithine concentration (open bars) and L-citrulline concentration (filled bars).** The cultivation was performed in CGXII minimal medium containing 20 g/L glucose, 1 mM IPTG, 750 μM L-arginine and 25 μg/L kanamycin. The amino acid concentrations in the supernatant were determined after the consumption of glucose. Values and error bars represent the mean and the standard error of triplicates.

**Figure 4:**
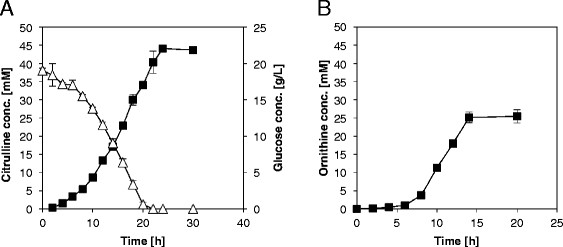
**Amino acid production by various** ***C. glutamicum strains.*** L-ornithine production by *C. glutamicum* CIT0(pVWEx1) (filled squares) **(A)** and L-citrulline accumulation (filled squares) and glucose consumption (open triangles) by strain CIT0(pVWEx1-*argFB*^fbr^) **(B).** The experiments were performed in CGXII minimal medium with 20 g/L glucose, 1 mM IPTG, 25 μg/L kanamycin and supplemented with 750 μM L-arginine. Values and error bars represent the mean and the standard error of triplicates.

To enable L-citrulline accumulation, two plasmids were constructed and used to transform *C. glutamicum* CIT0. While pVWEx1-*argF* only carries *argF* encoding L-ornithine carbamoyltransferase, pVWEx1-*argFB*^fbr^ in addition carries *argB*^fbr^ encoding feedback-resistant N-acetyl L-glutamate kinase (NAGK, EC 2.7.2.8). When grown in minimal medium with 2% glucose and 0.75 mM L-arginine *C. glutamicum* CIT0(pVWEx1-*argF*) grew to a higher OD than CIT0(pVWEx1) (Figure 2) and did not accumulate notable concentrations of L-citrulline. As opposed to CIT0(pVWEx1), CIT0(pVWEx1-*argF*) did not produce L-ornithine (Figure 3). By contrast, the combined overexpression of *argF* and *argB*^fbr^ entailed L-citrulline production and the respective strain was named CIT1. *C. glutamicum* CIT1 accumulated 44.1 ± 0.5 mM L-citrulline in minimal medium with 2% glucose (Figure 4).

When comparing the growth of *C. glutamicum* CIT0(pVWEx1) to that of CIT0(pVWEx1-*argF*), similar growth rates (0.37 ± 0.01 h^−1^ and 0.35 ± 0.04 h^−1^, respectively) were obtained, whereas L-citrulline formation by CIT0(pVWEx1-*argFB*^fbr^) was accompanied by a reduced growth rate (0.15 ± 0.01 h^−1^) (Figure 1). Moreover, the final OD_600_ of CIT0(pVWEx1-*argFB*^fbr^) was 20 ± 1 as compared to an OD_600_ of 26 ± 1 of CIT0(pVWEx1). By contrast, *C. glutamicum* CIT0(pVWEx1-*argF*) grew to a higher biomass concentration with a final OD_600_ of 35 ± 1. As shown in Figure 3, the lower growth rates of CIT0(pVWEx1) and CIT0(pVWEx1-*argFB*^fbr^) correlated inversely with the formation of the respective amino acids L-ornithine and L-citrulline, whereas *C. glutamicum* CIT0(pVWEx1-*argF*) reaches a higher final biomass and neither produces L-ornithine nor L-citrulline.

### Production of L-citrulline from alternative carbon sources

Due to the high demand of biotechnological processes of using complex sugar substrates derived from raw materials and industrial wastes, the L-citrulline producer strain CIT1 was enabled to utilize the alternative carbon sources starch (as an example of a high molecular weight carbohydrate), xylose, and glucosamine (as an example of a carbohydrates, derived from forestry and food industrial wastes).

To enable *C. glutamicum* CIT1 to consume starch, the gene *amyA* from *Streptomyces griseus* was overexpressed. The combined overexpression of *xylA* from *Xanthomonas campestris* and endogenous *xylB* allowed the utilization of xylose by *C. glutamicum* CIT1. The endogenous *nagB* was overpressed ectopically to facilitate the consumption of glucosamine. The resulting strains were tested for growth and L-citrulline production.

When cultured in CGXII medium supplemented 0.75 mM L-arginine all strains engineered for alternative carbon source consumption grew with their respective substrate (Table 1). The empty vector carrying strain CIT1(pEKEx3) neither grew in xylose or glucosamine minimal medium nor consumed these substrates. By contrast, the recombinant strain CIT1(pEKEx3-*xylAB*) grew in xylose minimal medium with a growth rate of 0.03 ± 0.01 h^−1^ and reached a final OD_600_ of 6 ± 1. In glucosamine minimal medium, *C. glutamicum* CIT1(pEKEx3-*nagB*) grew to a final OD_600_ of 3 ± 1 with a growth rate of 0.02 ± 0.01 h^−1^. In minimal medium containing 1% starch and 0.25% glucose as carbon sources, the empty vector harbouring strain CIT1(pEC-XT99A) formed roughly one third of the biomass as compared to *C. glutamicum* CIT1(pAmy). Growth of CIT1(pEC-XT99A) was slower (growth rate of 0.10 ± 0.01 h^−1^) than that of CIT1(pAmy) (growth rate of 0.21 ± 0.01 h^−1^). While strain CIT1(pEC-XT99A) only utilized glucose, but not starch, CIT1(pAmy) was able to consume both, glucose and starch.

**Figure 5:**
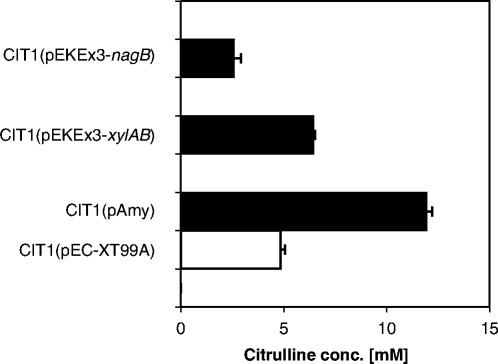
**L-citrulline concentration in the engineered strains after the consumption of the respective carbon source.** CIT1(pEC-XT99A), CIT1(pAmy) with 10 g/L soluble starch, 2,5 g/L glucose after 31 h. CIT1(pEKEx2-*xylAB*) with 15 g/L xylose after xylose consumption. CIT1(pEKEx3-*nagB*) with 10 g/L glucosamine after glucosamine consumption. Values and error bars represent the mean and the standard error of triplicates.

The strains engineered for utilization of xylose and glucosamine, respectively, also produced L-citrulline from these carbon sources (Figure 5). *C. glutamicum* CIT1(pEKEx3-*nagB*) accumulated 2.6 ± 0.3 mM L-citrulline which corresponds to a yield of 0.045 ± 0.002 g/g since glucosamine was utilized completely. Similarly, after complete utilization of xylose by *C. glutamicum* CIT1(pEKEx3-*xylAB*) 6.4 ± 0.1 mM L-citrulline accumulated corresponding to a yield of 0.075 ± 0.001 g per g xylose.

As the determination of the starch concentration by HPLC was not possible, residual starch content was assayed by the use of Lugols solution. However, as it is known that overexpression of *amyA* in *C. glutamicum* results in high molecular mass degradation products of starch, which remain in the medium and are not detectable by Lugols solution (Seibold et al. [2006]), the L-citrulline concentration was measured until no change in OD_600_, starch content and L-citrulline concentration was observed. The starch utilizing strain CIT1(pAmy) was able to produce 11.9 ± 0.5 mM L-citrulline which corresponds to a yield of 0.167 g/g.

## Discussion

*C. glutamicum* was engineered to accumulate L-citrulline as major product, both from glucose as well as from the alternative carbon sources starch, glucosamine and xylose.

Feedback insensitive N-acetyl L-glutamate kinase (encoded by *argB*^fbr^; (A49VM54V)) was required for production of L-citrulline since CIT0(pVWEx1-*argF*) did not produce L-citrulline, while CIT0(pVWEx1-*argFB*^fbr^) produced L-citrulline. It is unlikely that addition of L-arginine to CIT0(pVWEx1-*argF*) inhibited generation of L-ornithine, a precursor of L-citrulline, because strain CIT0(pVWEx1) produced L-ornithine when supplemented with L-arginine. However, it is possible that intracellular L-citrulline affects arginine biosynthesis. As overexpression of *argB*^fbr^ entailed L-citrulline formation, we assume that L-citrulline inhibits the NAGK of *C. glutamicum*, but this has not yet been described. As expected due to its structural similarity to L-arginine, L-citrulline inhibits NAGK of other microorganisms (Farago and Denes [1967]; Haas and Leisinger [1975]). In *Chlamydomonas reinhardtii*, NAGK is inhibited by several L-arginine structure analogs, including L-citrulline, however, inhibition was less pronounced than L-arginine inhibition (Farago and Denes [1967]). NAGK from *Pseudomonas aeruginosa* lost two thirds of its activity in the presence of 2.5 mM L-citrulline which was claimed to be too weak under physiologic conditions (Haas and Leisinger [1975]). However, it is conceivable that inhibition of NAGK by L-citrulline may play a role in recombinant *C. glutamicum* strains engineered for L-citrulline production, thus, possibly explaining the finding that L-citrulline production required overexpression *argB*^fbr^ encoding NAGK feedback resistant to L-arginine. Commensurate with this notion, simultaneous production of L-arginine and L-citrulline resulted from *argB*^fbr^ overexpression in a ∆*argR* background (Ikeda et al. [2009]). In this *argB*^fbr^ overexpressing strain, the ratio of L-citrulline to L-arginine was higher than by classically obtained strains, which solely contain native *argB* (Ikeda et al. [2009]). Currently, it remains to be studied if L-citrulline inhibits NAGK from *C. glutamicum* and if (some) variants feed-back resistant to L-arginine are also desensitized to L-citrulline.

Notably, about two fold more L-citrulline (about 7.7 g/L) was produced by strain CIT1 than L-ornithine was produced (about 3.3 g/L) by the isogenic strain CIT0(pVWEx1). Both, overexpression of *argF* and *argB*^fbr^ may have contributed to this effect. It is more likely that *argB*^fbr^ is responsible as L-arginine supplementation may have limited flux in the arginine biosynthesis pathway of strain CIT0(pVWEx1) especially in the beginning of the cultivation. In *C. glutamicum* CIT1, only feedback-resistant NAGK is present and additionally a gene dosage effect due ectopic overexpression of *argB*^fbr^ might have contributed to increase L-citrulline production.

Glucose, glucosamine, xylose, and starch were shown to be suitable substrates for the production of L-citrulline. Strain construction was based on previously established engineering strategies (Seibold et al. [2006]; Uhde et al. [2013]; Meiswinkel et al. [2013a]; Gopinath et al. [2011]). The achieved L-citrulline concentrations on these substrates were lower than with glucose as carbon source. However, L-citrulline production from xylose (6.44 ± 0.12 mM) by CIT1(pEKEx3-*xylAB*) was lower, but in a similar range as production of L-ornithine (19.6 ± 1.9 mM) and putrescine (15.1 ± 1.2 mM), respectively, from the same xylose concentration by the respective recombinant *C. glutamicum* strains (Meiswinkel et al. [2013a]). Similarly, product yields with glucosamine as carbon source were lower for L-citrulline (0.067 g/g) than for putrescine (0.112 g/g) (Uhde et al. [2013]). Unexpectedly and hitherto not understood, the growth rate (0.02 ± 0.01 h^−1^) and, thus, productivity by CIT1(pEKEx3-*nagB*) were very low. By contrast, a putrescine producing strain carrying pEKEx3-*nagB* showed only a slightly decreased growth rate (Uhde et al. [2013]).

*C. glutamicum* strains carrying pAMY co-utilized starch with glucose (Seibold et al. [2006]). Substrate co-utilization is observed with *C. glutamicum* WT as well as recombinant strains for almost all mixtures of carbon sources (Blombach and Seibold [2010]). A L-lysine producing strain carrying pAMY showed increased biomass formation by addition of 10 g/L starch to 10 g/L glucose, whereas L-lysine production increased only upon addition of higher starch concentrations (Seibold et al. [2006]).

In this study, the additional presence of starch increased the growth rate of CIT1 (from 0.15 to 0.21 h^−1^) as well as L-citrulline production. Production of L-citrulline by CIT1(pAMY) from a starch glucose mixture was higher (11.95 ± 0.48 mM) than that by the empty vector carrying control strain (4.83 ± 0.4 mM) demonstrating that starch contributed to production of L-citrulline. It has to be noted that starch cannot be utilized completely by *C. glutamicum* strains overexpressing the α-amylase gene *amyA* because high-molecular-weight carbohydrates are generated from starch and remain unutilized in the medium (Seibold et al. [2006]).

Taken together, production of L-citrulline as major product from glucose, starch, glucosamine, and xylose by recombinant *C. glutamicum* strains was achieved.

## Authors’ original submitted files for images

Below are the links to the authors’ original submitted files for images. Authors’ original file for figure 1 Authors’ original file for figure 2 Authors’ original file for figure 3 Authors’ original file for figure 4 Authors’ original file for figure 5
