# Supplementary figures and images for: L-citrulline production by metabolically engineered Corynebacterium glutamicum from glucose and alternative carbon sources
Source: AMB Express. 2014 Dec 10;4:85. doi: 10.1186/s13568-014-0085-0 (PMC4883986; doi:10.1186/s13568-014-0085-0)

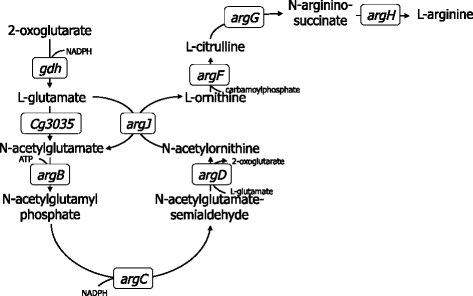

Supplement: Supplementary file 1 — Authors’ original file for figure 1 [file 13568_2014_85_MOESM1_ESM.gif]

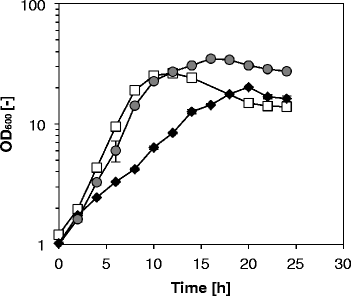

Supplement: Supplementary file 2 — Authors’ original file for figure 2 [file 13568_2014_85_MOESM2_ESM.gif]

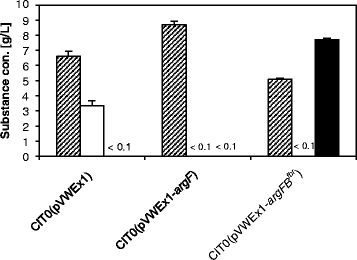

Supplement: Supplementary file 3 — Authors’ original file for figure 3 [file 13568_2014_85_MOESM3_ESM.gif]

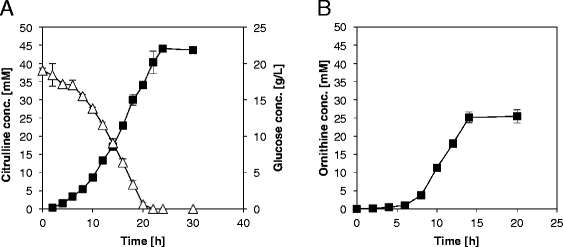

Supplement: Supplementary file 4 — Authors’ original file for figure 4 [file 13568_2014_85_MOESM4_ESM.gif]

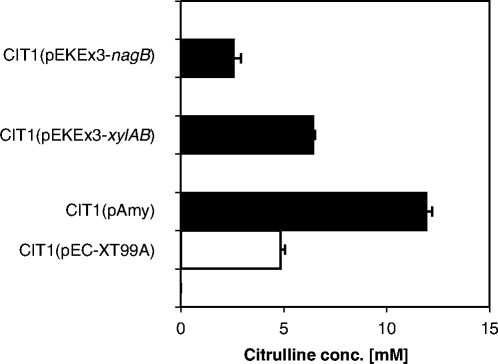

Supplement: Supplementary file 5 — Authors’ original file for figure 5 [file 13568_2014_85_MOESM5_ESM.gif]
